# Exponentiated power generalized Weibull power series family of distributions: Properties, estimation and applications

**DOI:** 10.1371/journal.pone.0230004

**Published:** 2020-03-20

**Authors:** Maha A. Aldahlan, Farrukh Jamal, Christophe Chesneau, Ibrahim Elbatal, Mohammed Elgarhy

**Affiliations:** 1 Department of Statistics, University of Jeddah, College of Science, Jeddah, Saudi Arabia; 2 Department of Statistics, Govt. S.A Postgraduate College Dera Nawab Sahib, Bahawalpur, Punjab, Pakistan; 3 Universitéde Caen, Caen, France; 4 Department of Mathematics and Statistics, College of Science Imam Mohammad Ibn Saud Islamic University (IMSIU), Riyadh, Saudi Arabia; 5 Department of Mathematical Statistics, Faculty of Graduate Studies for Statistical Research, Cairo University, Giza, Egypt; 6 Valley High Institute for Management Finance and Information Systems, Obour, Qaliubia, Egypt; Tongii University, CHINA

## Abstract

In this paper, we introduce the exponentiated power generalized Weibull power series (EPGWPS) family of distributions, obtained by compounding the exponentiated power generalized Weibull and power series distributions. By construction, the new family contains a myriad of new flexible lifetime distributions having strong physical interpretations (lifetime system, biological studies…). We discuss the characteristics and properties of the EPGWPS family, including its probability density and hazard rate functions, quantiles, moments, incomplete moments, skewness and kurtosis. The main vocation of the EPGWPS family remains to be applied in a statistical setting, and data analysis in particular. In this regard, we explore the estimation of the model parameters by the maximum likelihood method, with accuracy supported by a detailed simulation study. Then, we apply it to two practical data sets, showing the applicability and competitiveness of the EPGWPS models in comparison to some other well-reputed models.

## 1 Introduction

The Weibull distribution demonstrates adequate fits for most of the lifetime data, excepting those having empirical hazard rates with non-monotone shapes. Such data are often encountered in survival analysis, making the Weibull model useless to analyze them. Discussions in this regard can be found in [1]. The limitations of the Weibull distribution have motivated various generalizations and extensions, offering more flexible alternatives in terms of modelling. Among them, there are the extended Weibull distribution by [2], the new extended Weibull distribution by [3], the beta Weibull distribution by [4], the modified Weibull distribution by [5], the (P-A-L) extended Weibull distribution by [6], the additive Weibull distribution by [7], the generalized Weibull distribution by [8], the exponentiated Weibull distribution by [9], the Kumaraswamy Weibull distribution by [10] and the generalized modified Weibull distribution by [11].

More recently, a very flexible extension of the Weibull distribution was introduced by [12], called the exponentiated power generalized Weibull (EPGW) distribution. The cumulative distribution function (cdf) of the EPGW distribution with parameters *α*, *β*, λ and *μ* is given by

$$\begin{array}{c} G_{{}_{EPGW}}\left(x;\alpha ,\beta ,\lambda ,\mu \right)={\left[1-e^{1-{\left(1+\lambda x^{\mu }\right)}^{\alpha }}\right]}^{\beta },\;x>0, \end{array}$$ (1)

where λ > 0 is a scale parameter, and *μ*, *α*, *β* are shape parameters. For the sake of conciseness, we now set *φ* = (*α*, *β*, λ, *μ*). Then, the corresponding probability density function (pdf) and hazard rate function (hrf) are, respectively, given by

$$\begin{array}{c} g_{{}_{EPGW}}\left(x;\phi \right)=\beta \lambda \mu \alpha x^{\mu -1}{\left(1+\lambda x^{\mu }\right)}^{\alpha -1}e^{1-{\left(1+\lambda x^{\mu }\right)}^{\alpha }}{\left[1-e^{1-{\left(1+\lambda x^{\mu }\right)}^{\alpha }}\right]}^{\beta -1} \end{array}$$ (2)

and

$$\begin{array}{c} h_{{}_{EPGW}}\left(x;\phi \right)=\frac{\beta \lambda \mu \alpha x^{\mu -1}{\left(1+\lambda x^{\mu }\right)}^{\alpha -1}e^{1-{\left(1+\lambda x^{\mu }\right)}^{\alpha }}{\left[1-e^{1-{\left(1+\lambda x^{\mu }\right)}^{\alpha }}\right]}^{\beta -1}}{1-{\left[1-e^{1-{\left(1+\lambda x^{\mu }\right)}^{\alpha }}\right]}^{\beta }}. \end{array}$$ (3)

Among its interests, the EPGW distribution unifies several well-known lifetime distributions (such as the exponential, exponentiated exponential, Rayleigh, Burr type X, Weibull, exponentiated Weibull, Nadarajah-Haghighi, exponentiated Nadarajah-Haghighi and power generalized Weibull distributions), the corresponding hrf has flexible properties, showing decreasing, increasing, upside-down bathtub and bathtub shapes, if *β* is an integer, it corresponds to the distribution of the maximum lifetime of a random sample from the power generalized Weibull distribution and several practical investigations show that the EPGW model often gives better fits than other well-established generalized Weibull models. In this regard, we may refer the reader to the complete work of [12].

**Fig 1:**
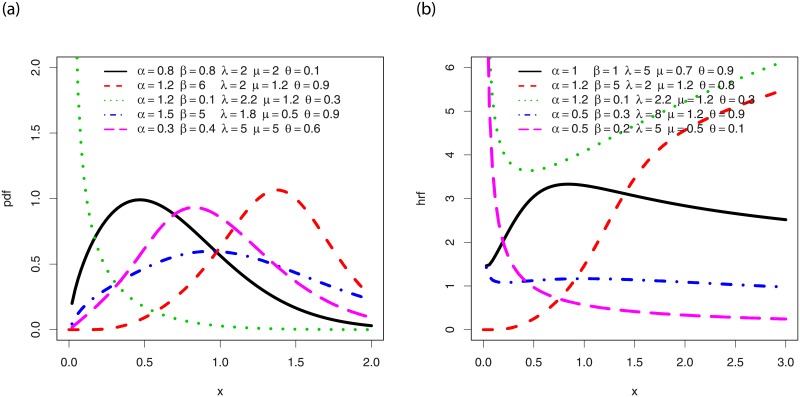
Plots of the (i) pdf of the EPGWG distribution and (ii) hrf of the EPGWG distribution, for various values of the parameters.

**Fig 2:**
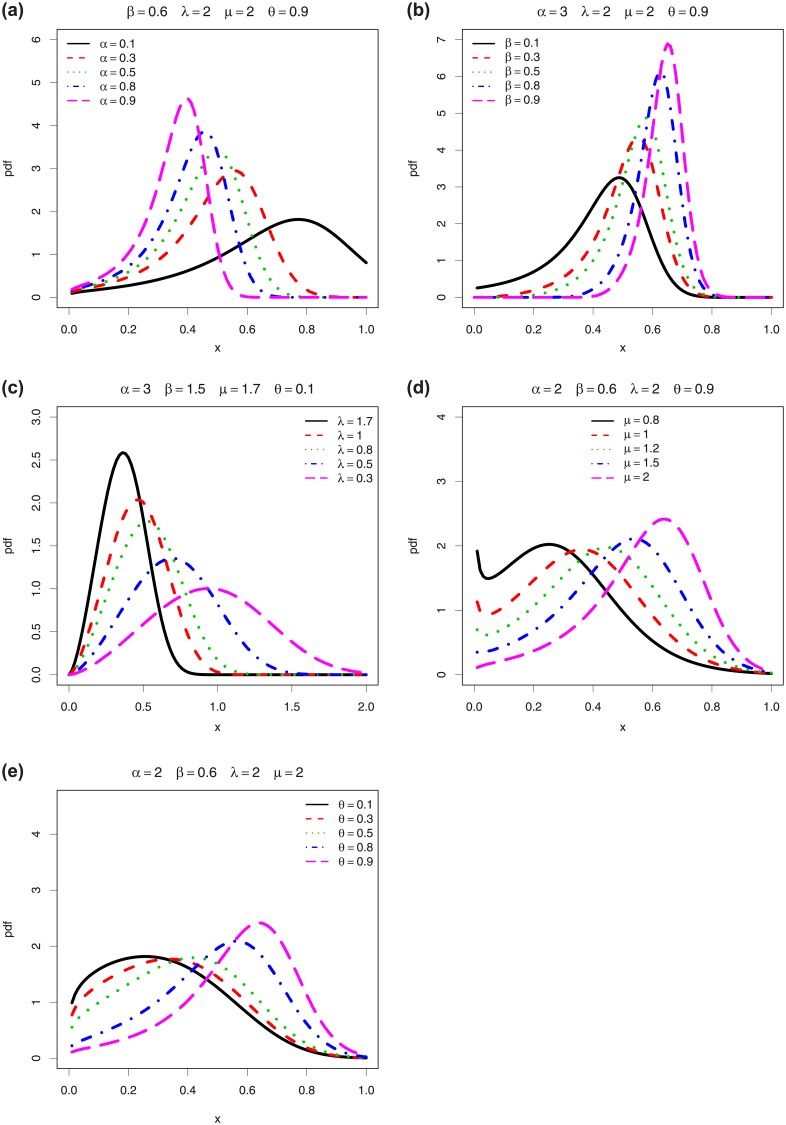
Plots of the pdf of the EPGWG distribution to better understand the roles of the parameters in its shape properties.

In this paper, motivated by these attractive properties, we aim to extend the EPGW distribution with the idea in mind to reach some new levels of flexibility, specially for the corresponding pdf and hrf (expecting more types of monotonic and non-monotonic curves in comparison to those of the pdf and hrf of the EPGW distribution (see [12, Figs 1 and 2])) and several crucial measures, such as moments, skewness and kurtosis. In this regard, we adopt the methodology introduced by [2] which consists in compounding continuous and power series distributions as introduced by [13], i.e., including geometric, Poisson, logarithmic and binomial distributions. The prime physical motivation behind this methodology is the modelling of a lifetime system depending on the random number of independent components with random lifetime. Thus, the number of component can be modeled by a zero-truncated discrete random variable *N* and the lifetime of the *i*-th component can be modelled by a positive continuous random variable *X*_*i*_. Then, the lifetime of the system can be modelled by either X_*_ = inf(*X*_1_, *X*_2_, …, *X*_*N*_) or X_**_ = sup(*X*_1_, *X*_2_, …, *X*_*N*_), depending on the structure of the components: series or parallel, respectively. Similar applications can be found in industrial and biological studies. From the statistical point of view, this compounding technique allows the construction of flexible families of distributions and has proved itself in different settings. We may refer the reader to the exponential power series family by [14], the Weibull power series family by [15], the generalized exponential power series family by [16], the extended Weibull power series family by [17], the Birnbaum- Saunders power series family by [18], the generalized modified Weibull power series family by [19], the exponentiated power Lindley power series family by [20], the inverse Weibull power series family by [21], the Burr-Weibull power series family by [22] and the complementary generalized power Weibull power series family by [23].

With the above arguments in mind, we introduce the exponentiated power generalized Weibull power series (EPGWPS) family obtained by compounding the EPGW and power series distributions. The new family contains a myriad of lifetime distributions as special members, such as the exponentiated power generalized Weibull geometric (EPGWG), exponentiated power generalized Weibull Poisson (EPGWP_*o*_), exponentiated power generalized Weibull binomial (EPGWB), exponentiated power generalized Weibull logarithmic (EPGWL), power generalized Weibull Poisson (PGWP_*o*_), power generalized Weibull geometric (PGWG), power generalized Weibull binomial (PGWB), and power generalized Weibull logarithmic (PGWL) distributions. The aim of this paper is to provide all the main features of the EPGWPS family, exploring both its mathematical and practical properties, with a focus on the EPGWG distribution. In particular, we highlight the attractive properties of the EPGWG model in a data analysis purpose. Two practical data sets are used in this regard.

The rest of the article is organized as follows. In Section 2, the EPGWPS family is introduced. In Section 3, we present some special members of the EPGWPS family, with a focus on the EPGWG distribution. Various mathematical and statistical properties of the new family are obtained in Section 4. Maximum likelihood estimates of the unknown parameters are presented in Section 5, as well as a simulation study. The EPGWG model is applied to two practical data sets in Section 6. We give some concluding remarks in Section 7.

## 2 The EPGWPS family of distributions

In this section, we define the EPGWPS family and present some of its special members of interest.

### 2.1 Definition

First of all, let us present the power series (PS) family of distributions. We consider a zero-truncated discrete random variable *N* having a power series probability mass function (pmf) given by

$$\begin{array}{c} P\left(N=n\right)=\frac{a_{n}\;\theta^{n}}{C(\theta )},\;n=1,2,\ldots , \end{array}$$ (4)

where *θ* > 0 (at least, it can belong to a more restrictive domain), *a*_*n*_ ≥ 0 depending only on *n*, and *C*(*θ*) is the normalization constant, i.e., $C\left(\theta \right)=\sum_{n=1}^{+\infty }a_{n}\theta^{n}$. We suppose that *C*(*θ*) is finite and its first, second and third derivatives with respect to *θ* are also finite and denoted by *C*′(*θ*), *C*′′(*θ*) and *C*′′′(*θ*), respectively. Then, the PS family is defined by the pmf given by (4) (see [13]). In particular, it includes the geometric, Poisson, logarithmic and binomial distributions, as detailed in Table 1.

**Table 1:** Useful quantities of some power series distributions.

Distribution	*a*_*n*_	*C*(*θ*)	*C*^−1^(*θ*)	Domain of *θ*
Geometric	1	*θ*(1 − *θ*)^−1^	*θ*(1 + *θ*)^−1^	(0, 1)
Poisson	(*n*!)^−1^	*e*^*θ*^ − 1	log(*θ* + 1)	(0, + ∞)
Binomial	(mn)	(*θ* + 1)^*m*^ − 1	(*θ* + 1)^1/*m*^ − 1	(0, + ∞)
Logarithmic	*n*^−1^	−log(1 − *θ*)	1 − *e*^−*θ*^	(0, 1)

Now, let us consider a sequence of independent and identically distributed random variable *X*_1_, *X*_2_, … and X_**_ = sup(*X*_1_, *X*_2_, …, *X*_*N*_). Then, based on (1) and (4), the conditional cumulative distribution function (cdf) of X_**_∣*N* = *n* is given by

$$\begin{array}{c} G_{{}_{X_{**}|N=n}}\left(x;\phi \right)={\left[G_{{}_{EPGW}}\left(x;\phi \right)\right]}^{n}={\left[1-e^{{}^{1-{\left(1+\lambda x^{\mu }\right)}^{\alpha }}}\right]}^{\beta n},\;x>0. \end{array}$$ (5)

One can notice that it is the cdf of the EPGW distribution with parameters *α*, *βn*, λ and *μ*. Then, the EPGWPS family is defined by the cdf of X_**_ given by

$$\begin{array}{cc} F(x;\phi ,\theta ) & =\sum_{n=1}^{\infty }\frac{a_{n}\theta^{n}}{C(\theta )}G_{{}_{X_{**}|N=n}}\left(x;\phi \right)=\frac{C[\theta G_{{}_{EPGW}}\left(x;\phi \right)]}{C(\theta )}\\ & =\frac{C\{\theta {\left[1-e^{{}^{1-{\left(1+\lambda x^{\mu }\right)}^{\alpha }}}\right]}^{\beta }\}}{C(\theta )},\;x>0. \end{array}$$ (6)

Based on this last expression, the corresponding pdf and hrf are, respectively, given by

$$\begin{array}{cc} & f\left(x;\phi ,\theta \right)=\theta g_{{}_{EPGW}}\left(x;\phi \right)\frac{C^{\prime }[\theta G_{{}_{EPGW}}\left(x;\phi \right)]}{C(\theta )}\\ & =\theta \beta \lambda \mu \alpha x^{\mu -1}{\left(1+\lambda x^{\mu }\right)}^{\alpha -1}e^{{}^{1-{\left(1+\lambda x^{\mu }\right)}^{\alpha }}}{\left[1-e^{{}^{1-{\left(1+\lambda x^{\mu }\right)}^{\alpha }}}\right]}^{\beta -1}\frac{C^{\prime }\{\theta {\left[1-e^{{}^{1-{\left(1+\lambda x^{\mu }\right)}^{\alpha }}}\right]}^{\beta }\}}{C(\theta )} \end{array}$$ (7)

and

$$\begin{array}{cc} h(x;\phi ,\theta ) & =\theta g_{{}_{EPGW}}\left(x;\phi \right)\frac{C^{\prime }[\theta G_{{}_{EPGW}}\left(x;\phi \right)]}{C\left(\theta \right)-C[\theta G_{{}_{EPGW}}\left(x;\phi \right)]}\\ & =\theta \beta \lambda \mu \alpha x^{\mu -1}{\left(1+\lambda x^{\mu }\right)}^{\alpha -1}e^{{}^{1-{\left(1+\lambda x^{\mu }\right)}^{\alpha }}}{\left[1-e^{{}^{1-{\left(1+\lambda x^{\mu }\right)}^{\alpha }}}\right]}^{\beta -1}\times \\ & \frac{C^{\prime }\{\theta {\left[1-e^{{}^{1-{\left(1+\lambda x^{\mu }\right)}^{\alpha }}}\right]}^{\beta }\}}{C\left(\theta \right)-C\{\theta {\left[1-e^{{}^{1-{\left(1+\lambda x^{\mu }\right)}^{\alpha }}}\right]}^{\beta }\}}. \end{array}$$ (8)

This new family is quite flexible because it contains a plethora of well-established lifetime distributions and some new ones. In this regard, Table 2 lists some of them derived from the EPGWPS family.

**Table 2:** Some distributions derived from the EPGWPS family (with varying λ and *θ*).

Name	*μ*	*α*	*β*	Family	References
ENHPS	1	−	−	exponentiated Nadarajah-Haghighi power series	New
NHPS	1	−	1	Nadarajah-Haghighi power series	New
PGWPS	−	−	1	power generalized Weibull power series	[23]
EWPS	−	1	−	exponentiated Weibull power series	[16]
WPS	−	1	1	Weibull power series	[15]
EExPS	1	1	−	generalized exponential power series	[16]
ExPS	1	1	1	exponential power series	[16]
BPS	2	1	−	Burr type X power series	New

### 2.2 Special members of the EPGWPS family

In this section, we present special members of the EPGWPS family based on the discrete distribution presented in Table 1. Thus, we introduce the exponentiated power generalized Weibull geometric (EPGWG), exponentiated power generalized Weibull Poisson (EPGWP_*o*_), exponentiated power generalized Weibull binomial (EPGWB) and exponentiated power generalized Weibull logarithmic (EPGWL) distributions.

**EPGWG distribution**: The EPGWG distribution arises by taking *a*_*n*_ = 1 and *C*(*θ*) = *θ*(1 − *θ*)^−1^ with *θ* ∈ (0, 1). By using the cdf given by (6), the EPGWG distribution is defined by the following cdf:
  $$\begin{array}{c} F\left(x;\phi ,\theta \right)=\frac{\left(1-\theta \right){\left[1-e^{1-{\left(1+\lambda x^{\mu }\right)}^{\alpha }}\right]}^{\beta }}{1-\theta {\left[1-e^{1-{\left(1+\lambda x^{\mu }\right)}^{\alpha }}\right]}^{\beta }},\;x>0. \end{array}$$ (9)
  One can remark that it is also a member of the Marshall-Olkin family defined with the EPGW distribution as baseline (see [2]). Also, by using (7) and (8), the corresponding pdf and hrf are, respectively, given by
  $$\begin{array}{c} f\left(x;\phi ,\theta \right)=\frac{\left(1-\theta \right)\beta \lambda \mu \alpha x^{\mu -1}{\left(1+\lambda x^{\mu }\right)}^{\alpha -1}e^{1-{\left(1+\lambda x^{\mu }\right)}^{\alpha }}{\left[1-e^{1-{\left(1+\lambda x^{\mu }\right)}^{\alpha }}\right]}^{\beta -1}}{{\left\{1-\theta {\left[1-e^{1-{\left(1+\lambda x^{\mu }\right)}^{\alpha }}\right]}^{\beta }\right\}}^{2}} \end{array}$$ (10)
  and
  $$\begin{array}{c} h\left(x;\phi ,\theta \right)=\frac{\left(1-\theta \right)\beta \lambda \mu \alpha x^{\mu -1}{\left(1+\lambda x^{\mu }\right)}^{\alpha -1}e^{1-{\left(1+\lambda x^{\mu }\right)}^{\alpha }}{\left[1-e^{1-{\left(1+\lambda x^{\mu }\right)}^{\alpha }}\right]}^{\beta -1}}{\left\{1-\theta {\left[1-e^{1-{\left(1+\lambda x^{\mu }\right)}^{\alpha }}\right]}^{\beta }\}\{1-{\left[1-e^{1-{\left(1+\lambda x^{\mu }\right)}^{\alpha }}\right]}^{\beta }\right\}}. \end{array}$$ (11)
**EPGWP_*o*_ distribution**: The EPGWP_*o*_ distribution arises by taking *a*_*n*_ = (*n*!)^−1^ and *C*(*θ*) = *e*^*θ*^ − 1, with *θ* > 0. By using the cdf given by (6), the PGWP_*o*_ distribution is defined by the following cdf:
  $$\begin{array}{c} F\left(x;\phi ,\theta \right)=\frac{e^{\theta {\left[1-e^{1-{\left(1+\lambda x^{\mu }\right)}^{\alpha }}\right]}^{\beta }}-1}{e^{\theta }-1},\;x>0. \end{array}$$
  Also, from (7) and (8), the corresponding pdf and hrf are, respectively, given by
  $$\begin{array}{cc} f(x;\phi ,\theta ) & =\frac{\theta \beta \lambda \mu \alpha x^{\mu -1}{\left(1+\lambda x^{\mu }\right)}^{\alpha -1}}{e^{\theta }-1}e^{1-{\left(1+\lambda x^{\mu }\right)}^{\alpha }}{\left[1-e^{1-{\left(1+\lambda x^{\mu }\right)}^{\alpha }}\right]}^{\beta -1}\times \\ & e^{\theta {\left[1-e^{1-{\left(1+\lambda x^{\mu }\right)}^{\alpha }}\right]}^{\beta }} \end{array}$$
  and
  $$\begin{array}{cc} h(x;\phi ,\theta ) & =\frac{\theta \beta \lambda \mu \alpha x^{\mu -1}{\left(1+\lambda x^{\mu }\right)}^{\alpha -1}}{e^{\theta }-e^{\theta {\left[1-e^{1-{\left(1+\lambda x^{\mu }\right)}^{\alpha }}\right]}^{\beta }}}e^{1-{\left(1+\lambda x^{\mu }\right)}^{\alpha }}{\left[1-e^{1-{\left(1+\lambda x^{\mu }\right)}^{\alpha }}\right]}^{\beta -1}\times \\ & e^{\theta {\left[1-e^{1-{\left(1+\lambda x^{\mu }\right)}^{\alpha }}\right]}^{\beta }}. \end{array}$$
**EPGWB distribution**: The EPGWB distribution arises by taking an=(mn) and *C*(*θ*) = (*θ* + 1)^*m*^ − 1, with *θ* > 0 and *m* ≥ *n*. Hence, from (6), the cdf of the PGWP_*o*_ distribution can be expressed as
  $$\begin{array}{c} F\left(x;\phi ,\theta \right)=\frac{{\left\{\theta {\left[1-e^{1-{\left(1+\lambda x^{\mu }\right)}^{\alpha }}\right]}^{\beta }+1\right\}}^{m}-1}{{\left(\theta +1\right)}^{m}-1},\;x>0. \end{array}$$
  Also, from (7) and (8), the corresponding pdf and hrf are, respectively, given by
  $$\begin{array}{cc} f(x;\phi ,\theta ) & =m\theta \beta \lambda \mu \alpha x^{\mu -1}{\left(1+\lambda x^{\mu }\right)}^{\alpha -1}e^{1-{\left(1+\lambda x^{\mu }\right)}^{\alpha }}{\left[1-e^{1-{\left(1+\lambda x^{\mu }\right)}^{\alpha }}\right]}^{\beta -1}\\ & \times \frac{{\left\{\theta {\left[1-e^{1-{\left(1+\lambda x^{\mu }\right)}^{\alpha }}\right]}^{\beta }+1\right\}}^{m-1}}{{\left(\theta +1\right)}^{m}-1} \end{array}$$
  and
  $$\begin{array}{cc} h(x;\phi ,\theta ) & =m\theta \beta \lambda \mu \alpha x^{\mu -1}{\left(1+\lambda x^{\mu }\right)}^{\alpha -1}e^{1-{\left(1+\lambda x^{\mu }\right)}^{\alpha }}{\left[1-e^{1-{\left(1+\lambda x^{\mu }\right)}^{\alpha }}\right]}^{\beta -1}\\ & \times \frac{{\left\{\theta {\left[1-e^{1-{\left(1+\lambda x^{\mu }\right)}^{\alpha }}\right]}^{\beta }+1\right\}}^{m-1}}{{\left(\theta +1\right)}^{m}-{\left\{\theta {\left[1-e^{1-{\left(1+\lambda x^{\mu }\right)}^{\alpha }}\right]}^{\beta }+1\right\}}^{m}}. \end{array}$$
**EPGWL distribution**: The EPGWL distribution arises by taking *a*_*n*_ = *n*^−1^ and *C*(*θ*) = −log(1 − *θ*), with *θ* ∈ (0, 1). Hence, from (6), the EPGWL distribution has the following cdf:
  $$\begin{array}{c} F\left(x;\phi ,\theta \right)=\frac{\log\{1-\theta {\left[1-e^{1-{\left(1+\lambda x^{\mu }\right)}^{\alpha }}\right]}^{\beta }\}}{\log(1-\theta )},\;x>0. \end{array}$$
  Also, from (7) and (8), the corresponding pdf and hrf are, respectively, given by
  $$\begin{array}{c} f\left(x;\phi ,\theta \right)=\frac{\theta \beta \lambda \mu \alpha x^{\mu -1}{\left(1+\lambda x^{\mu }\right)}^{\alpha -1}e^{1-{\left(1+\lambda x^{\mu }\right)}^{\alpha }}{\left[1-e^{1-{\left(1+\lambda x^{\mu }\right)}^{\alpha }}\right]}^{\beta -1}}{\log(1-\theta )\{\theta {\left[1-e^{1-{\left(1+\lambda x^{\mu }\right)}^{\alpha }}\right]}^{\beta }-1\}} \end{array}$$
  and
  $$\begin{array}{c} h\left(x;\phi ,\theta \right)=\frac{\theta \beta \lambda \mu \alpha x^{\mu -1}{\left(1+\lambda x^{\mu }\right)}^{\alpha -1}e^{1-{\left(1+\lambda x^{\mu }\right)}^{\alpha }}{\left[1-e^{1-{\left(1+\lambda x^{\mu }\right)}^{\alpha }}\right]}^{\beta -1}}{\left\{\theta {\left[1-e^{1-{\left(1+\lambda x^{\mu }\right)}^{\alpha }}\right]}^{\beta }-1\}\log\{\frac{1-\theta }{1-\theta {\left[1-e^{1-{\left(1+\lambda x^{\mu }\right)}^{\alpha }}\right]}^{\beta }}\right\}}. \end{array}$$

## 3 On the EPGWG distribution

First fo all, we recall that the EPGWG distribution is defined with the cdf, pdf and hrf given by (9), (10) and (11), respectively. Preliminaries works show attractive properties for this distribution in terms of flexibility of the related functions, thats why we put the light on it in this section. Let us investigate the asymptotic properties of *f*(*x*;*ϕ*, *θ*) and *h*(*x*;*ϕ*, *θ*). When *x* → 0, we have

$$\begin{array}{c} F\left(x;\phi ,\theta \right)∼\left(1-\theta \right)\alpha^{\beta }\lambda^{\beta }x^{\mu \beta },\;f\left(x;\phi ,\theta \right)∼\left(1-\theta \right)\alpha^{\beta }\lambda^{\beta }\mu \beta x^{\mu \beta -1} \end{array}$$

and

$$\begin{array}{c} h\left(x;\phi ,\theta \right)∼\left(1-\theta \right)\alpha^{\beta }\lambda^{\beta }\mu \beta x^{\mu \beta -1}. \end{array}$$

So

$$\begin{array}{c} \lim_{x\to 0}f\left(x;\phi ,\theta \right)=\lim_{x\to 0}h\left(x;\phi ,\theta \right)=\{\begin{array}{cccc} & +\infty & & \text{if}\;\mu \beta <1,\\ & \left(1-\theta \right)\alpha^{\beta }\lambda^{\beta } & & \text{if}\;\mu \beta =1,\\ & 0 & & \text{if}\;\mu \beta >1. \end{array} \end{array}$$

In this case, we see that the values of *μ* and *β* are determinant in the asymptotic properties of *f*(*x*;*ϕ*, *θ*) and *h*(*x*;*ϕ*, *θ*). Also, when *x* → + ∞, we have

$$\begin{array}{c} 1-F\left(x;\phi ,\theta \right)∼\frac{1}{1-\theta }\beta e^{1-{\left(1+\lambda x^{\mu }\right)}^{\alpha }},\;f\left(x;\phi ,\theta \right)∼\frac{1}{1-\theta }\beta \lambda^{\alpha }\mu \alpha x^{\mu \alpha -1}e^{1-{\left(1+\lambda x^{\mu }\right)}^{\alpha }} \end{array}$$

and

$$\begin{array}{c} \;h\left(x;\phi ,\theta \right)∼\lambda^{\alpha }\mu \alpha x^{\mu \alpha -1}. \end{array}$$

Hence

$$\begin{array}{c} \lim_{x\to +\infty }f\left(x;\phi ,\theta \right)=0,\;\lim_{x\to +\infty }h\left(x;\phi ,\theta \right)=\{\begin{array}{cccc} & 0 & & \text{if}\;\mu \alpha <1,\\ & \lambda^{\alpha } & & \text{if}\;\mu \alpha =1,\\ & +\infty & & \text{if}\;\mu \alpha >1. \end{array} \end{array}$$

Here, *μ* and *α* are determinant in the asymptotic properties of *f*(*x*;*ϕ*, *θ*) and *h*(*x*;*ϕ*, *θ*). Naturally, the curves of *f*(*x*;*ϕ*, *θ*) and *h*(*x*;*ϕ*, *θ*) can take various forms depending on the values of the parameters. In this regard, the critical points of these functions can be of interest. A critical point for *f*(*x*;*ϕ*, *θ*) is solution of the following non-linear equation: {log[*f*(*x*;*ϕ*, *θ*)]}′ = 0 according to *x*, where, after some algebra,

$$\begin{array}{cc} {\left\{\log[f(x;\phi ,\theta )]\right\}}^{\prime } & =\left(\mu -1\right)\frac{1}{x}+\left(\alpha -1\right)\lambda \mu \frac{x^{\mu -1}}{1+\lambda x^{\mu }}-\lambda \alpha \mu x^{\mu -1}{\left(1+\lambda x^{\mu }\right)}^{\alpha -1}\\ & +\left(\beta -1\right)\lambda \alpha \mu \frac{x^{\mu -1}{\left(1+\lambda x^{\mu }\right)}^{\alpha -1}e^{1-{\left(1+\lambda x^{\mu }\right)}^{\alpha }}}{1-e^{1-{\left(1+\lambda x^{\mu }\right)}^{\alpha }}}\\ & +2\theta \beta \lambda \mu \alpha \frac{x^{\mu -1}{\left(1+\lambda x^{\mu }\right)}^{\alpha -1}e^{1-{\left(1+\lambda x^{\mu }\right)}^{\alpha }}{\left[1-e^{1-{\left(1+\lambda x^{\mu }\right)}^{\alpha }}\right]}^{\beta -1}}{1-\theta {\left[1-e^{1-{\left(1+\lambda x^{\mu }\right)}^{\alpha }}\right]}^{\beta }}. \end{array}$$

The degree of complexity of this equation is high; only a mathematical software can be used to provide a numerical evaluation of a critical point. Also, its nature depends on the sign of {log[*f*(*x*;*ϕ*, *θ*)]}′′ taken at this point, which requires a similar numerical treatment. Similarly, a critical point for *h*(*x*;*ϕ*, *θ*) is solution of the following non-linear equation: {log[*h*(*x*;*ϕ*, *θ*)]}′ = 0, where

$$\begin{array}{cc} {\left\{\log[h(x;\phi ,\theta )]\right\}}^{\prime } & =\left(\mu -1\right)\frac{1}{x}+\left(\alpha -1\right)\lambda \mu \frac{x^{\mu -1}}{1+\lambda x^{\mu }}-\lambda \alpha \mu x^{\mu -1}{\left(1+\lambda x^{\mu }\right)}^{\alpha -1}\\ & +\left(\beta -1\right)\lambda \alpha \mu \frac{x^{\mu -1}{\left(1+\lambda x^{\mu }\right)}^{\alpha -1}e^{1-{\left(1+\lambda x^{\mu }\right)}^{\alpha }}}{1-e^{1-{\left(1+\lambda x^{\mu }\right)}^{\alpha }}}\\ & +2\theta \beta \lambda \mu \alpha \frac{x^{\mu -1}{\left(1+\lambda x^{\mu }\right)}^{\alpha -1}e^{1-{\left(1+\lambda x^{\mu }\right)}^{\alpha }}{\left[1-e^{1-{\left(1+\lambda x^{\mu }\right)}^{\alpha }}\right]}^{\beta -1}}{1-\theta {\left[1-e^{1-{\left(1+\lambda x^{\mu }\right)}^{\alpha }}\right]}^{\beta }}\\ & +\left(1-\theta \right)\beta \lambda \mu \alpha \frac{x^{\mu -1}{\left(1+\lambda x^{\mu }\right)}^{\alpha -1}e^{1-{\left(1+\lambda x^{\mu }\right)}^{\alpha }}{\left[1-e^{1-{\left(1+\lambda x^{\mu }\right)}^{\alpha }}\right]}^{\beta -1}}{\left\{1-\theta {\left[1-e^{1-{\left(1+\lambda x^{\mu }\right)}^{\alpha }}\right]}^{\beta }\}\{1-{\left[1-e^{1-{\left(1+\lambda x^{\mu }\right)}^{\alpha }}\right]}^{\beta }\right\}}. \end{array}$$

Again, the considered non-linear equation is too massive to expect an analytical solution, only a numerical solution can be derived; a mathematical software can help in this regard. The same remark holds for the nature of such a critical point which requires the calculus of {log[*h*(*x*;*ϕ*, *θ*)]}′′ taken at this point.

A comprehensive study on the shapes of *f*(*x*;*ϕ*, *θ*) and *h*(*x*;*ϕ*, *θ*) can be performed by a simple graphical approach. Hence, Fig 1(i) shows plots of *f*(*x*;*ϕ*, *θ*) for various values of the parameters and Fig 1(ii) shows the same for *h*(*x*;*ϕ*, *θ*).

From Fig 1(i), we observe that *f*(*x*;*ϕ*, *θ*) can be decreasing and has left skewed, right skewed and (near) symmetrical shapes, with various degree of skewness and kurtosis. Also, Fig 1(ii) reveals a wide panel of shapes for *h*(*x*;*ϕ*, *θ*), including decreasing, increasing, (near) constant and non-monotonic shapes of various kinds (reverse J, tilde, U (bathtub) and reverse U).

We now highlight the individual role of the parameters in the shapes of *f*(*x*;*ϕ*, *θ*) in Fig 2.

In particular, for the considered values, we see that *α* and λ have great effects on the kurtosis, *β* mainly impacts the mode, *μ* can produce tilde shapes and impact the skewness, and *θ* has an influence on the nature of the skewness and can round the top of the curve. In particular, Figs 1 and 2 reveal that the pdf and hrf of the EPGWG distribution has more rich curvatures forms in comparison to the pdf and hrf of the former EPGW distribution (see [12, Figs 1 and 2]). This makes the EPGWG distribution very attractive to model various kinds of lifetime data.

## 4 Properties

In this section, some mathematical and statistical properties of the EPGWPS family are discussed.

### 4.1 On the EPGWPS and EPGW distributions

Here, we discuss some immediate results on the EPGWPS family. First fo all, let us notice that the cdf of X_*_ = inf(*X*_1_, *X*_2_, …, *X*_*N*_) is given by

$$\begin{array}{cc} F_{{}_{X_{*}}}\left(x;\phi ,\theta \right) & =1-\frac{C[\theta -\theta G_{{}_{EPGW}}\left(x;\phi \right)]}{C(\theta )}=1-\frac{C\{\theta -\theta {\left[1-e^{{}^{1-{\left(1+\lambda x^{\mu }\right)}^{\alpha }}}\right]}^{\beta }\}}{C(\theta )},\;x>0. \end{array}$$

The following result shows the relation existing between X_*_ and the EPGW family.

**Proposition 4.1** *Let* $G_{{}_{EPGW}}^{-1}\left(x;\phi \right)$ *be the quantile function corresponding to* *G*_*EPGW*_ (*x*; *ϕ*). *Then, the random variable*

$$\begin{array}{c} Y=G_{{}_{EPGW}}^{-1}\left[1-G_{{}_{EPGW}}\left(X_{*};\phi \right);\phi \right] \end{array}$$

*has the cdf of the EPGWPS family*.

**Proof**: For any x∈R, the cdf of *Y* is given by

$$\begin{array}{cc} F_{Y}\left(x;\phi ,\theta \right) & =P\left(Y\leq x\right)=P\{G_{{}_{EPGW}}^{-1}\left[1-G_{{}_{EPGW}}\left(X_{*};\phi \right);\phi \right]\leq x\}\\ & =P\{X_{*}\geq G_{{}_{EPGW}}^{-1}\left[1-G_{{}_{EPGW}}\left(x;\phi \right);\phi \right]\}\\ & =1-F_{{}_{X_{*}}}\{G_{{}_{EPGW}}^{-1}\left[1-G_{{}_{EPGW}}\left(x;\phi \right);\phi \right]\}=\frac{C[\theta G_{{}_{EPGW}}\left(x;\phi \right)]}{C(\theta )}. \end{array}$$

We recognize the cdf of the EPGWPS family, ending the proof of Proposition 4.1.

A simple link between the EPGWPS family and the EPGW distribution is determined below.

**Proposition 4.2** *Let d* = min{*n* ∈ *N*: *a*_*n*_ > 0}. *Then, when θ* → 0^+^, *the EPGWPS family defined with the parameters α, β*, λ, *μ and θ has for limiting case the EPGW distribution with parameter α, βd*, λ *and μ*.

**Proof**: The limit of the cdf of the EPGWPS family when *θ* → 0^+^ is determined as follows:

$$\begin{array}{cc} \lim_{\theta \to 0^{+}}F\left(x;\phi ,\theta \right) & =\lim_{\theta \to 0^{+}}\frac{C[\theta G_{{}_{EPGW}}\left(x;\phi \right)]}{C(\theta )}=\lim_{\theta \to 0^{+}}\frac{\sum_{n=1}^{+\infty }a_{n}\theta^{n}{\left[G_{{}_{EPGW}}\left(x;\phi \right)\right]}^{n}}{\sum_{n=1}^{+\infty }a_{n}\theta^{n}}\\ & =\lim_{\theta \to 0^{+}}\frac{a_{d}{\left[G_{{}_{EPGW}}\left(x;\phi \right)\right]}^{d}+\sum_{n=d+1}^{+\infty }a_{n}\theta^{n-d}{\left[G_{{}_{EPGW}}\left(x;\phi \right)\right]}^{n}}{a_{d}+\sum_{n=d+1}^{+\infty }a_{n}\theta^{n-d}}\\ & ={\left[G_{{}_{EPGW}}\left(x;\phi \right)\right]}^{d}={\left[1-e^{1-{\left(1+\lambda x^{\mu }\right)}^{\alpha }}\right]}^{\beta d}. \end{array}$$

We thus obtain the cdf of the EPGW distribution with parameter *α*, *βd*, λ and *μ*. This ends the proof of Proposition 4.2.

The following result is about a stochastic order involving the EPGWPS family and EPGW distribution.

**Proposition 4.3** *Let R and S be two random variables such that R follows the EPGW distribution and S has the cdf of the EPGWPS family, both with identical parameters ϕ. Then S is greater to R in likelihood ratio order, i.e., the ratio function of the corresponding pdfs (the one of S over the one of R) is increasing*.

**Proof**: Let *f*(*x*;*ϕ*, *θ*) be the cdf of *S* and *g*_*EPGW*_ (*x*; *ϕ*) be the pdf of *R*. Then, owing to (7), we have

$$\begin{array}{cc} & \frac{f(x;\phi ,\theta )}{g_{{}_{EPGW}}\left(x;\phi \right)}=\theta \frac{C^{\prime }[\theta G_{{}_{EPGW}}\left(x;\phi \right)]}{C(\theta )}, \end{array}$$

which is increasing according to *x* as composition of increasing functions. This proves Proposition 4.3.

The following result highlights an important representation of the pdf of the EPGWPS family.

**Proposition 4.4** *The pdf of the EPGWPS family can be expressed as an infinite number of linear combination (mixture) of pdfs of order statistics of the EPGW distribution*.

**Proof**: Upon differentiation according to *x* of the first expression of *F*(*x*;*ϕ*, *θ*) in (6), almost surely, the pdf of the EPGWPS family is given by

$$\begin{array}{cc} f(x;\phi ,\theta ) & =\sum_{n=1}^{+\infty }\frac{a_{n}\theta^{n}}{C(\theta )}g_{{}_{X_{**}|N=n}}\left(x;\phi \right), \end{array}$$ (12)

where $g_{{}_{X_{**}|N=n}}\left(x;\phi \right)$ is the pdf of the EPGW distribution with parameters *α*, *βn*, λ and *μ*, i.e.,

$$\begin{array}{c} g_{{}_{X_{**}|N=n}}\left(x;\phi \right)=n\beta \lambda \mu \alpha x^{\mu -1}{\left(1+\lambda x^{\mu }\right)}^{\alpha -1}e^{1-{\left(1+\lambda x^{\mu }\right)}^{\alpha }}{\left[1-e^{1-{\left(1+\lambda x^{\mu }\right)}^{\alpha }}\right]}^{n\beta -1}. \end{array}$$

This proves Proposition 4.4.

Hence, by virtue of Proposition 4.4, we can obtain some mathematical properties of the EPGWPS family by using those of the EPGW distribution.

### 4.2 Quantile function

The quantile function of the EPGWPS family is the function *Q*(*y*;*ϕ*, *θ*), *y* ∈ (0, 1), such that *F*(*Q*(*y*;*ϕ*, *θ*), *ϕ*, *θ*) = *y*. By using (6), after some algebra, we get

$$\begin{array}{c} Q\left(y;\phi ,\theta \right)=G_{{}_{EPGW}}^{-1}\{\frac{C^{-1}\left[yC\left(\theta \right)\right]}{\theta };\phi \},\;y\in \left(0,1\right), \end{array}$$

where

$$\begin{array}{c} G_{{}_{EPGW}}^{-1}\left(y;\phi \right)={\left[\frac{1}{\lambda }\{{\left[1-\log(1-y^{1/\beta })\right]}^{1/\alpha }-1\}\right]}^{1/\mu },\;y\in \left(0,1\right) \end{array}$$

and *C*^−1^(*θ*) is the inverse function of *C*(*θ*).

In particular, for the EPGWG distribution, we have *C*(*θ*) = *θ*(1 − *θ*)^−1^ and *C*^−1^(*θ*) = *θ*(1 + *θ*)^−1^, implying that

$$\begin{array}{c} Q\left(y;\phi ,\theta \right)={\left[\frac{1}{\lambda }\left\{{\left[1-\log(1-{\left[y{\left(1-\theta +y\theta \right)}^{-1}\right]}^{1/\beta })\right]}^{1/\alpha }-1\right\}\right]}^{1/\mu },\;y\in \left(0,1\right). \end{array}$$

The *q*-quantiles are defined by *x*_*q*_(*ϕ*, *θ*) = *Q*(*q*;*ϕ*, *θ*), including the median defined by *M*_*e*_(*ϕ*, *θ*) = *x*_0.5_(*ϕ*, *θ*). Also, the quantile function is of importance to define some measures of skewness and kurtosis, which will be the object of the next section, and to generate values from the EPGWPS family.

### 4.3 Skewness and kurtosis based on quantiles

We now present some useful measures of skewness and kurtosis of the EPGWPS family based on the quantile function. To evaluate the skewness of the EPGWPS family, we can use Galton skewness introduced by [24] and defined by

$$\begin{array}{c} B\left(\phi ,\theta \right)=\frac{Q(1/4;\phi ,\theta )+Q(3/4;\phi ,\theta )-2Q(1/2;\phi ,\theta )}{Q(3/4;\phi ,\theta )-Q(1/4;\phi ,\theta )}. \end{array}$$

Then, if *B*(*ϕ*, *θ*)<0, the distribution is left skewed, if *B*(*ϕ*, *θ*)>0, it is right skewed and if *B*(*ϕ*, *θ*) = 0, it is symmetrical. Also, a well-established kurtosis measure is the Moors kurtosis introduced by [25] and defined by

$$\begin{array}{c} M\left(\phi ,\theta \right)=\frac{Q(7/8;\phi ,\theta )-Q(5/8;\phi ,\theta )+Q(3/8;\phi ,\theta )-Q(1/8;\phi ,\theta )}{Q(6/8;\phi ,\theta )-Q(2/8;\phi ,\theta )}. \end{array}$$

A high value of *M*(*ϕ*, *θ*) rather indicates a heavy tail for the distribution and a small value of *M*(*ϕ*, *θ*) rather indicates a light tail.

**Fig 3:**
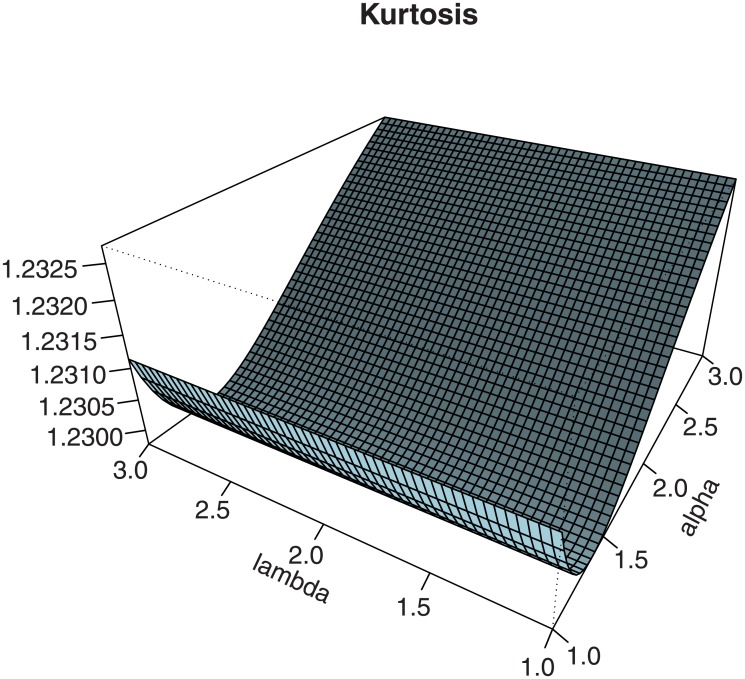
Plots for the (i) Galton skewness and (ii) Moors kurtosis of the EPGWG distribution, for 1 < *α*, λ < 3, *β* = 3, *μ* = 2 and *θ* = 0.4.

**Fig 4:**
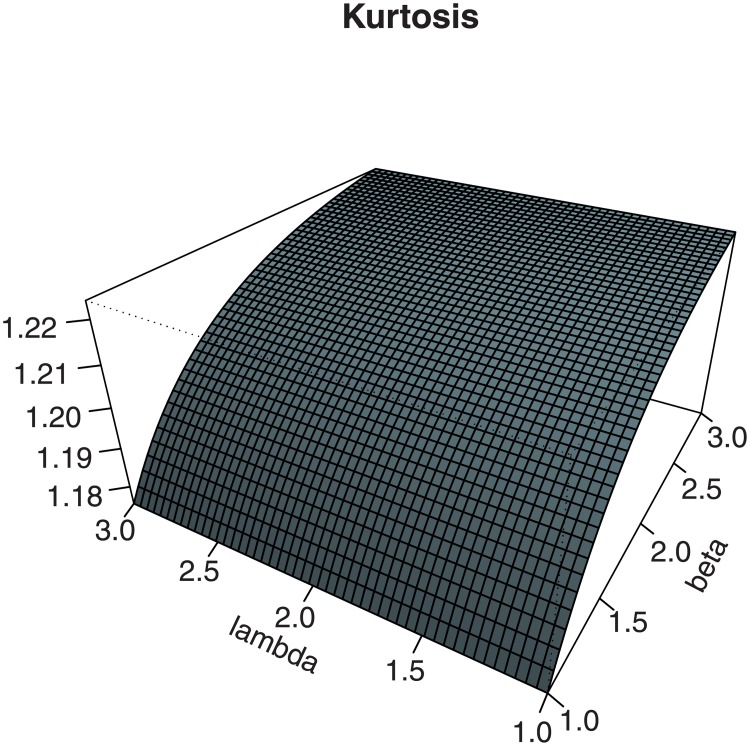
Plots for the (i) Galton skewness and (ii) Moors kurtosis of the EPGWG distribution, for 1 < *β*, λ < 3, *α* = 3, *μ* = 2 and *θ* = 0.1.

Figs 3 and 4 investigate graphically the comportment of these two measures in the context of the EPGWG distribution, according to the values of the parameters.

We observe various monotonic and non-monotonic shapes, with possible negative and positive values for the Galton skewness, showing the flexibility of these measures. This completes, in some sense, the wide panel of skewness and kurtosis already noticed in Figs 1 and 2.

### 4.4 Moments

The moments plays an important role in any statistical analysis. They allow to measure crucial features of a distribution (dispersion, skewness, kurtosis…). Here, we investigate different kinds of moments of the EPGWPS family, with some related measures. Based on (7), the *r*-th raw moment of the EPGWPS family is given by

$$\begin{array}{cc} \mu_{r}^{\prime }\left(\phi ,\theta \right) & =\int_{-\infty }^{+\infty }x^{r}f\left(x;\phi ,\theta \right)dx\\ & =\int_{0}^{+\infty }x^{r}\theta \beta \lambda \mu \alpha x^{\mu -1}{\left(1+\lambda x^{\mu }\right)}^{\alpha -1}e^{{}^{1-{\left(1+\lambda x^{\mu }\right)}^{\alpha }}}{\left[1-e^{{}^{1-{\left(1+\lambda x^{\mu }\right)}^{\alpha }}}\right]}^{\beta -1}\times \\ & \frac{C^{\prime }\{\theta {\left[1-e^{{}^{1-{\left(1+\lambda x^{\mu }\right)}^{\alpha }}}\right]}^{\beta }\}}{C(\theta )}dx. \end{array}$$

This integral is complex; the use of a mathematical software is necessary to obtain a numerical evaluation of it.

Alternatively, we can provide a series expansion of $\mu_{r}^{\prime }\left(\phi ,\theta \right)$ by using (13). Indeed, we have

$$\begin{array}{cc} \mu_{r}^{\prime }\left(\phi ,\theta \right) & =\sum_{n=1}^{+\infty }\frac{a_{n}\theta^{n}}{C(\theta )}\mu_{{}_{EPGW};r,n}^{\prime }\left(\phi \right), \end{array}$$ (13)

where $\mu_{{}_{EPGW};r,n}^{\prime }\left(\phi \right)$ denotes the *r*-th raw moment of the EPGW distribution with parameters *α*, *βn*, λ and *μ*, which is given by [12, Equation (12)], i.e.,

μEPGW;r,n′(ϕ)=βnλ-r/μ∑i=0+∞∑j=0+∞(-1)i+jej+1(j+1)[r-μ(i-α)]/(αμ)(βn-1j)(r/μi)Γ(r-μ(i-α)αμ,j+1),

where $\Gamma \left(a,x\right)=\int_{x}^{+\infty }t^{a-1}e^{-t}dt$ denotes the complementary incomplete gamma function.

Based on the raw moments, we can express the central moments *μ*_*r*_(*ϕ*, *θ*) and cumulants *κ*_*r*_(*ϕ*, *θ*) of the EPGWPS family as, respectively,

μr(ϕ,θ)=∑m=0r(rm)(-1)mμ1′(ϕ,θ)mμr-m′(ϕ,θ)

and

κr(ϕ,θ)=μr′(ϕ,θ)-∑m=1r-1(r-1m-1)κm(ϕ,θ)μr-m′(ϕ,θ),

where $\kappa_{1}\left(\phi ,\theta \right)=\mu_{1}^{\prime }\left(\phi ,\theta \right)$. The variance is given by *σ*^2^(*ϕ*, *θ*) = *μ*_2_(*ϕ*, *θ*). Also, we can defined some skewness and kurtosis measures based on moments, as the skewness coefficient defined by *SK*(*ϕ*, *θ*) = *μ*_3_(*ϕ*, *θ*)/*σ*^3^(*ϕ*, *θ*) = *κ*_3_(*ϕ*, *θ*)/*κ*_2_(*ϕ*, *θ*)^3/2^ and the kurtosis coefficient defined by *KU*(*ϕ*, *θ*) = *μ*_4_(*ϕ*, *θ*)/*σ*^4^(*ϕ*, *θ*) = *κ*_4_(*ϕ*, *θ*)/*κ*_2_(*ϕ*, *θ*)^2^ + 3, respectively.

As illustration, Table 3 presents some numerical values of the first four moments, variance, skewness and kurtosis of the EPGW distribution for some values of the parameters. We thus see the flexibility of these measures according to the values of the parameters. In particular, negative and positive values for the skewness are observed, as well as small (with one negative) and high values for the kurtosis.

**Table 3:** The numerical values of the first four moments, variance, skewness and kurtosis of the EPGW distribution for some values of the parameters.

(*α*, *β*, λ, *μ*, *θ*)	$\mu_{1}^{\prime }\left(\phi ,\theta \right)$	$\mu_{2}^{\prime }\left(\phi ,\theta \right)$	$\mu_{3}^{\prime }\left(\phi ,\theta \right)$	$\mu_{4}^{\prime }\left(\phi ,\theta \right)$	*σ*^2^(*ϕ*, *θ*)	*SK*(*ϕ*, *θ*)	*KU*(*ϕ*, *θ*)
(0.5, 0.5, 0.5, 0.5, 0.5)	70.0015	28790.48	17528965	12505353679	23890.26	3.2954	13.4583
(1.5, 0.5, 0.5, 0.5, 0.5)	2.0382	19.0176	345.2229	9805.441	14.8630	4.2908	32.9325
(1.5, 1.5, 0.5, 0.5, 0.5)	4.5018	50.9162	992.0219	28944.68	30.6496	2.8691	14.5714
(1.5, 1.5, 1.5, 0.5, 0.5)	0.5002	0.6285	1.3607	4.4116	0.3783	2.8691	26.2835
(1.5, 1.5, 1.5, 1.5, 0.5)	0.6784	0.5483	0.5002	0.5012	0.0880	0.3339	259.3242
(2, 1.5, 1.5, 1.5, 0.5)	0.5236	0.3204	0.2181	0.1610	0.0462	0.1844	436.9716
(2, 5, 1.5, 1.5, 0.5)	0.7057	0.5292	0.4182	0.3462	0.0311	0.1371	2214.396
(2, 5, 2, 1.5, 0.5)	0.5825	0.3606	0.2352	0.1607	0.0212	0.1371	2702.193
(2, 5, 2, 2, 0.5)	0.7570	0.5825	0.4551	0.3606	0.0094	-0.2526	29021.35
(2, 5, 2, 2, 0.9)	0.8404	0.7140	0.6125	0.5301	0.0076	-0.6825	61187.26
(2, 10, 0.2, 0.3, 0.9)	764.0262	953257.2	1785251287	4.8988 × 10^12^	369521.2	2.1916	3.4390
(5, 10, 0.2, 0.3, 0.9)	12.5788	216.9296	4782.063	131187	58.7030	1.2818	-5.2072

### 4.5 Incomplete moments

The incomplete moments find numerous applications in lifetime models. They allow to define important quantities, such as the mean residual lifetime and mean inactivity time functions, as well as mean deviations, Bonferroni and Lorenz curves. Here, we provide expressions for the incomplete moments of the EPGWPS family. Based on (7), the *r*-th incomplete moment of the EPGWPS family takes the form

$$\begin{array}{cc} m_{r}\left(t;\phi ,\theta \right) & =\int_{-\infty }^{t}x^{r}f\left(x;\phi ,\theta \right)dx\\ & =\int_{0}^{t}x^{r}\theta \beta \lambda \mu \alpha x^{\mu -1}{\left(1+\lambda x^{\mu }\right)}^{\alpha -1}e^{{}^{1-{\left(1+\lambda x^{\mu }\right)}^{\alpha }}}{\left[1-e^{{}^{1-{\left(1+\lambda x^{\mu }\right)}^{\alpha }}}\right]}^{\beta -1}\times \\ & \frac{C^{\prime }\{\theta {\left[1-e^{{}^{1-{\left(1+\lambda x^{\mu }\right)}^{\alpha }}}\right]}^{\beta }\}}{C(\theta )}dx. \end{array}$$

Due to its complexity, only a mathematical software can give numerical value for this integral.

Alternatively, as for the *r*-th raw moment, we can provide a series expansion of $\mu_{r}^{\prime }\left(t;\phi ,\theta \right)$ by using (13) as

$$\begin{array}{cc} m_{r}\left(t;\phi ,\theta \right) & =\sum_{n=1}^{+\infty }\frac{a_{n}\theta^{n}}{C(\theta )}m_{{}_{EPGW};r,n}\left(t;\phi \right), \end{array}$$

where *m*_*EPGW*;*r*,*n*_(*t*;*ϕ*) denotes the *r*-th incomplete moment of the EPGW distribution with parameters *α*, *βn*, λ and *μ*, which is given by [12, Section 5], i.e.,

mEPGW;r,n(t,ϕ)=βnλ-r/μ∑i=0+∞∑j=0+∞(-1)i+jej+1(j+1)[r-μ(i-α)]/(αμ)(βn-1j)(r/μi)×{Γ(r-μ(i-α)αμ,j+1)-Γ(r-μ(i-α)αμ,(j+1)(1+λtμ)α)}.

In particular, thanks to *m*_1_(*t*;*ϕ*, *θ*), if a random variable *X* has the cdf of the EPGWPS family, then we can derive the mean deviations about the mean $\mu_{1}^{\prime }\left(\phi ,\theta \right)$ and the mean deviations about the median *M*(*ϕ*, *θ*) are, respectively defined by

$$\delta_{1}\left(\phi ,\theta \right)=E[|X-\mu_{1}^{\prime }\left(\phi ,\theta \right)|]=2\mu_{1}^{\prime }\left(\phi ,\theta \right)F\left(\mu_{1}^{\prime };\phi ,\theta \right)-2m_{1}\left(\mu_{1}^{\prime }\left(\phi ,\theta \right);\phi ,\theta \right)$$

and

$$\delta_{2}\left(\phi ,\theta \right)=E[|X-M_{e}\left(\phi ,\theta \right)|]=\mu_{1}^{\prime }\left(\phi ,\theta \right)-2m_{1}\left(M\left(\phi ,\theta \right);\phi ,\theta \right).$$

Also, the Lorenz and Bonferroni curves can be expressed via *m*_1_(*t*;*ϕ*, *θ*) as, respectively,

$$\begin{array}{c} L\left(p;\phi ,\theta \right)=\frac{m_{1}[x_{p}\left(\phi ,\theta \right);\phi ,\theta ]}{\mu_{1}^{\prime }\left(\phi ,\theta \right)},\;B\left(p;\phi ,\theta \right)=\frac{m_{1}[x_{p}\left(\phi ,\theta \right);\phi ,\theta ]}{p\mu_{1}^{\prime }\left(\phi ,\theta \right)},\;p\in \left(0,1\right). \end{array}$$ (14)

These curves finds applications in many areas, such as economics, reliability, medicine and insurance. A nice survey in this regard can be found in [26].

**Fig 5:**
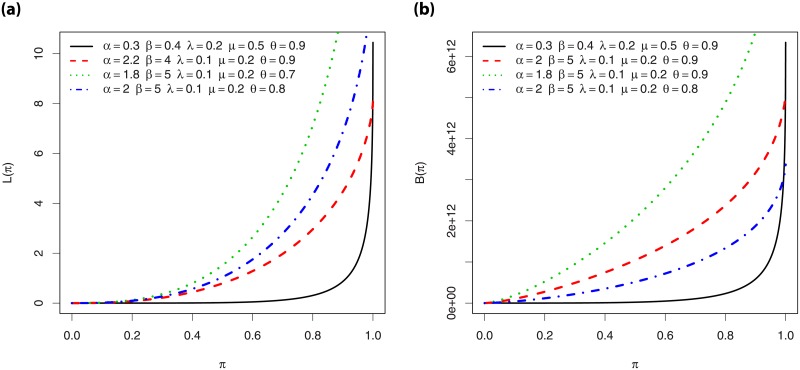
Plots of the (i) Lorenz curve of the EPGWG distribution and (ii) Bonferroni curve of the EPGWG distribution, for various values of the parameters.

Fig 5 displays these curve for the EPGWG distribution. More or less convex curves are observed.

## 5 Estimation and inference

In this section, we investigate the maximum likelihood estimates (MLEs) of the parameters of the EPGWPS family, from complete samples only.

### 5.1 Method

Let *x*_1_, …, *x*_*n*_ be a random sample of size *n* from the EPGWPS family and *ψ* = (*α*, *β*, λ, *μ*, *θ*)^*T*^. Then, the total likelihood and log-likelihood functions for *ψ* are, respectively, given by

$$\begin{array}{cc} L(\psi ) & =\theta^{n}\beta^{n}\lambda^{n}\mu^{n}\alpha^{n}\times \\ & \prod_{i=1}^{n}x_{i}^{\mu -1}{\left(1+\lambda x_{i}^{\mu }\right)}^{\alpha -1}e^{{}^{1-{\left(1+\lambda x_{i}^{\mu }\right)}^{\alpha }}}{\left[1-e^{{}^{1-{\left(1+\lambda x_{i}^{\mu }\right)}^{\alpha }}}\right]}^{\beta -1}\frac{C^{\prime }\{\theta {\left[1-e^{{}^{1-{\left(1+\lambda x_{i}^{\mu }\right)}^{\alpha }}}\right]}^{\beta }\}}{C(\theta )} \end{array}$$

and

$$\begin{array}{cc} \ell (\psi ) & =n\log\left(\theta \right)+n\log\left(\beta \right)+n\log\left(\lambda \right)+n\log\left(\mu \right)+n\log\left(\alpha \right)+\left(\mu -1\right)\sum_{i=1}^{n}\log\left(x_{i}\right)\\ & +\left(\alpha -1\right)\sum_{i=1}^{n}\log(1+\lambda x_{i}^{\mu })+n-\sum_{i=1}^{n}{\left(1+\lambda x_{i}^{\mu }\right)}^{\alpha }+\left(\beta -1\right)\sum_{i=1}^{n}\log[1-e^{1-{\left(1+\lambda x_{i}^{\mu }\right)}^{\alpha }}]\\ & +\sum_{i=1}^{n}\log[C^{\prime }\{\theta {\left[1-e^{1-{\left(1+\lambda x_{i}^{\mu }\right)}^{\alpha }}\right]}^{\beta }\}]-n\log\left[C\left(\theta \right)\right]. \end{array}$$

The MLE of *ψ*, say $\hat{\psi }$, is obtained by solving the nonlinear system *U*_*n*_(*ψ*) = 0, where $U_{n}\left(\psi \right)={\left(\frac{\partial \ell (\psi )}{\partial \alpha },\frac{\partial \ell (\psi )}{\partial \beta },\frac{\partial \ell (\psi )}{\partial \lambda },\frac{\partial \ell (\psi )}{\partial \mu },\frac{\partial \ell (\psi )}{\partial \theta }\right)}^{T}$. Let us now express the components of *U*_*n*_(*ψ*). For the sake of conciseness, let us set, for *i* = 1, 2, …, *n*,

$$\begin{array}{c} \Omega_{i}=\frac{C^{\prime \prime }\{\theta {\left[1-e^{{}^{1-{\left(1+\lambda x_{i}^{\mu }\right)}^{\alpha }}}\right]}^{\beta }\}}{C^{\prime }\{\theta {\left[1-e^{1-{\left(1+\lambda x_{i}^{\mu }\right)}^{\alpha }}\right]}^{\beta }\}}. \end{array}$$

Then, we have

$$\begin{array}{cc} \frac{\partial \ell (\psi )}{\partial \alpha } & =\frac{n}{\alpha }+\sum_{i=1}^{n}\log(1+\lambda x_{i}^{\mu })-\sum_{i=1}^{n}{\left(1+\lambda x_{i}^{\mu }\right)}^{\alpha }\log(1+\lambda x_{i}^{\mu })\\ & +\left(\beta -1\right)\sum_{i=1}^{n}\frac{{\left(1+\lambda x_{i}^{\mu }\right)}^{\alpha }\log(1+\lambda x_{i}^{\mu })e^{1-{\left(1+\lambda x_{i}^{\mu }\right)}^{\alpha }}}{1-e^{{}^{1-{\left(1+\lambda x_{i}^{\mu }\right)}^{\alpha }}}}\\ & +\theta \beta \sum_{i=1}^{n}\Omega_{i}{\left[1-e^{1-{\left(1+\lambda x_{i}^{\mu }\right)}^{\alpha }}\right]}^{\beta -1}{\left(1+\lambda x_{i}^{\mu }\right)}^{\alpha }\log(1+\lambda x_{i}^{\mu })e^{1-{\left(1+\lambda x_{i}^{\mu }\right)}^{\alpha }}, \end{array}$$

$$\begin{array}{cc} \frac{\partial \ell (\psi )}{\partial \beta } & =\frac{n}{\beta }+\sum_{i=1}^{n}\log[1-e^{{}^{1-{\left(1+\lambda x_{i}^{\mu }\right)}^{\alpha }}}]+\theta \sum_{i=1}^{n}\Omega_{i}{\left[1-e^{1-{\left(1+\lambda x_{i}^{\mu }\right)}^{\alpha }}\right]}^{\beta }\times \\ & \log[1-e^{1-{\left(1+\lambda x_{i}^{\mu }\right)}^{\alpha }}], \end{array}$$

$$\begin{array}{cc} \frac{\partial \ell (\psi )}{\partial \lambda } & =\frac{n}{\lambda }+\left(\alpha -1\right)\sum_{i=1}^{n}\frac{x_{i}^{\mu }}{1+\lambda x_{i}^{\mu }}-\alpha \sum_{i=1}^{n}x_{i}^{\mu }{\left(1+\lambda x_{i}^{\mu }\right)}^{\alpha -1}\\ & +\alpha \left(\beta -1\right)\sum_{i=1}^{n}\frac{x_{i}^{\mu }{\left(1+\lambda x_{i}^{\mu }\right)}^{\alpha -1}e^{{}^{1-{\left(1+\lambda x_{i}^{\mu }\right)}^{\alpha }}}}{1-e^{1-{\left(1+\lambda x_{i}^{\mu }\right)}^{\alpha }}}\\ & +\theta \beta \alpha \sum_{i=1}^{n}\Omega_{i}{\left[1-e^{1-{\left(1+\lambda x_{i}^{\mu }\right)}^{\alpha }}\right]}^{\beta -1}e^{1-{\left(1+\lambda x_{i}^{\mu }\right)}^{\alpha }}x_{i}^{\mu }{\left(1+\lambda x_{i}^{\mu }\right)}^{\alpha -1}, \end{array}$$

$$\begin{array}{cc} \frac{\partial \ell (\psi )}{\partial \mu } & =\frac{n}{\mu }+\sum_{i=1}^{n}\log\left(x_{i}\right)+\left(\alpha -1\right)\lambda \sum_{i=1}^{n}\frac{x_{i}^{\mu }\log\left(x_{i}\right)}{1+\lambda x_{i}^{\mu }}-\alpha \lambda \sum_{i=1}^{n}x_{i}^{\mu }\log\left(x_{i}\right){\left(1+\lambda x_{i}^{\mu }\right)}^{\alpha -1}\\ & +\alpha \left(\beta -1\right)\lambda \sum_{i=1}^{n}\frac{x_{i}^{\mu }\log\left(x_{i}\right){\left(1+\lambda x_{i}^{\mu }\right)}^{\alpha -1}e^{{}^{1-{\left(1+\lambda x_{i}^{\mu }\right)}^{\alpha }}}}{1-e^{{}^{1-{\left(1+\lambda x_{i}^{\mu }\right)}^{\alpha }}}}\\ & +\theta \beta \alpha \lambda \sum_{i=1}^{n}\Omega_{i}{\left[1-e^{1-{\left(1+\lambda x_{i}^{\mu }\right)}^{\alpha }}\right]}^{\beta -1}e^{1-{\left(1+\lambda x_{i}^{\mu }\right)}^{\alpha }}\log\left(x_{i}\right)x_{i}^{\mu }{\left(1+\lambda x_{i}^{\mu }\right)}^{\alpha -1} \end{array}$$

and

$$\begin{array}{c} \frac{\partial \ell (\psi )}{\partial \theta }=\frac{n}{\theta }+\sum_{i=1}^{n}\Omega_{i}{\left[1-e^{1-{\left(1+\lambda x_{i}^{\mu }\right)}^{\alpha }}\right]}^{\beta }-n\frac{C^{\prime }\left(\theta \right)}{C(\theta )}. \end{array}$$

Since $\hat{\psi }$ has not a closed form, a mathematical software can be used for a numerical evaluation. For interval estimation and hypothesis tests on *ψ*, the corresponding observed information matrix is required. Here, the observed information matrix is given by *I*(*ψ*) = {−*I*_*rs*_}_(*r*, *s*)∈{1, …,5}^2^_, where, by denoting *ψ*_*r*_ the *r*-th component of *ψ*,

$$\begin{array}{c} I_{rs}=\frac{\partial^{2}\ell \left(\psi \right)}{\partial \psi_{r}\partial \psi_{s}}. \end{array}$$

Applying the well-established theory on the MLEs, the asymptotic multivariate normal distribution $N_{5}\left(\psi ,I{\left(\hat{\psi }\right)}^{-1}\right)$ can be used to construct approximate confidence intervals for the parameters, among others. In particular, by denoting ${\hat{\psi }}_{r}$ the *r*-th component of $\hat{\psi }$, an asymptotic confidence interval for *ψ*_*r*_ at the level 100(1 − *γ*)% is given by

$$ACI_{r}=({\hat{\psi }}_{r}-z_{1-\gamma /2}\sqrt{\hat{I_{rr}}},{\hat{\psi }}_{r}+z_{1-\gamma /2}\sqrt{\hat{I_{rr}}}),$$

where $\hat{I_{rr}}$ is the *r*-th diagonal element of $I{\left(\hat{\psi }\right)}^{-1}$ and *z*_1−*γ*/2_ is the (1 − *γ*/2)-quantile of the standard normal distribution N(0,1).

### 5.2 Simulation study

Here, in the context of the EPGWG model, we perform a Monte-Carlo simulation study to check the accuracy of the MLE $\hat{\psi }$; it is expected to be as close as possible to *ψ*, specially when the size of the sample is consequent, which is guaranteed by the well-know convergent properties of the related estimator. Thus, for several values of *n*, we generate *N* = 1000 random samples of size *n* from the EPGWG distribution via the use of the corresponding quantile function, under the following sets of parameters, with order (*α*, *β*, λ, *μ*, *θ*):

Set1: (1.5, 1.5, 1.5, 1.5, 0.5), Set2: (1.5, 1.5, 1.5, 1.5, 0.2), Set3: (2, 1.5, 1.5, 1.5, 0.5),
Set4: (2, 2, 1.5, 1.5, 0.5), Set5: (2, 2, 2, 1.5, 0.5), Set6: (2, 2, 2, 0.5, 0.5).

Then, the MLEs are determined for each sample. For each *n*, from the *N* obtained MLEs, we determine the mean square error (MSE) for $\hat{\psi }$. The numerical results are collected in Tables 4 and 5.

**Table 4:** The MLEs and MSEs of the EPGWG model for Set1, Set2 and Set3.

*n*	Set1: (1.5, 1.5, 1.5, 1.5, 0.5)		Set2: (1.5, 1.5, 1.5, 1.5, 0.2)		Set3: (2, 1.5, 1.5, 1.5, 0.5)	
	MLE	MSE	MLE	MSE	MLE	MSE
100	1.5075	0.0235	1.5143	0.0231	2.0391	0.0353
	1.5458	0.0328	1.5257	0.0368	1.5179	0.0377
	1.5428	0.0488	1.5259	0.0357	1.5654	0.0526
	1.6677	0.2562	1.6306	0.2565	1.6438	0.3659
	0.5101	0.0122	0.2101	0.0029	0.5355	0.0153
200	1.5094	0.0113	1.5083	0.0128	2.0136	0.0231
	1.5093	0.0166	1.5119	0.0162	1.5275	0.0273
	1.5130	0.0173	1.5210	0.0266	1.5226	0.0232
	1.5506	0.0949	1.5588	0.0993	1.6139	0.1870
	0.5129	0.0072	0.2047	0.0011	0.5104	0.0081
300	1.5054	0.0100	1.4971	0.0082	2.0038	0.0124
	1.5021	0.0122	1.5114	0.0122	1.4905	0.0134
	1.4995	0.0140	1.4988	0.0152	1.4826	0.0185
	1.5224	0.0639	1.5418	0.0610	1.4992	0.0847
	0.5098	0.0050	0.2015	0.0007	0.5098	0.0042
500	1.5060	0.0059	1.5061	0.0036	1.9962	0.0062
	1.5096	0.0090	1.5032	0.0057	1.5101	0.0087
	1.5096	0.0070	1.5100	0.0079	1.4964	0.0069
	1.5367	0.0501	1.5185	0.0298	1.5343	0.0470
	0.5045	0.0027	0.2021	0.0003	0.4997	0.0022
1000	1.5079	0.0024	1.5049	0.0025	1.9987	0.0037
	1.4917	0.0038	1.5016	0.0037	1.5039	0.0037
	1.5044	0.0039	1.5066	0.0036	1.4999	0.0042
	1.4905	0.0174	1.5101	0.0177	1.5143	0.0192
	0.5087	0.0014	0.2016	0.0002	0.5007	0.0012

**Table 5:** The MLEs and MSEs of the EPGWG model for Set4, Set5 and Set6.

*n*	Set4: (2, 2, 1.5, 1.5, 0.5)		Set5: (2, 2, 2, 1.5, 0.5)		Set6: (2, 2, 2, 0.5, 0.5)	
	MLE	MSE	MLE	MSE	MLE	MSE
100	2.0165	0.0438	2.0097	0.0433	2.0121	0.0392
	2.0257	0.0773	2.0177	0.0669	2.0709	0.0925
	1.5072	0.0442	2.0092	0.0801	2.0531	0.0852
	1.6103	0.2858	1.7146	0.7465	0.5082	1.5117
	0.5387	0.0313	0.6632	1.4415	0.5310	0.0124
200	1.9855	0.0202	2.0140	0.0230	2.0056	0.0221
	2.0343	0.0315	2.0180	0.0410	2.0313	0.0385
	1.4900	0.0226	2.0236	0.0368	2.0204	0.0329
	1.5733	0.0843	1.6361	0.3257	0.5525	0.0374
	0.5012	0.0083	0.5407	0.0334	0.5105	0.0032
300	1.9791	0.0103	1.9900	0.0104	2.0115	0.0168
	2.0361	0.0220	2.0227	0.0267	1.9943	0.0288
	1.4897	0.0111	1.9909	0.0225	2.0038	0.0262
	1.5742	0.0663	1.6055	0.1819	0.5163	0.0232
	0.4935	0.0043	0.5059	0.0085	0.4979	0.0022
500	1.9965	0.0073	2.0246	0.0072	1.9957	0.0059
	2.0100	0.0166	1.9974	0.0127	2.0201	0.0135
	1.4963	0.0076	2.0364	0.0132	2.0039	0.0112
	1.5289	0.0484	1.5247	0.0608	0.5232	0.0083
	0.5029	0.0030	0.5211	0.0071	0.5055	0.0015
1000	2.0024	0.0044	2.0016	0.0039	2.0011	0.0049
	2.0054	0.0061	1.9953	0.0081	2.0050	0.0090
	1.5043	0.0040	1.9956	0.0067	2.0000	0.0088
	1.5136	0.0146	1.5040	0.0365	0.5089	0.0051
	0.5027	0.0018	0.5090	0.0032	0.5007	0.0009

From these tables, for all the considered sets of parameters, we see that the MSEs decrease as the sample sizes increases, as expected; this is consistent with the theoretical properties of convergence of the MLEs.

## 6 Applications

In this section, the EPGWG distribution is used as model to analyze two practical data sets given below.

**D1** The first data set is obtained from [27]. The data are as follows: 3.70, 2.74, 2.73, 2.50, 3.60, 3.11, 3.27, 2.87, 1.47, 3.11, 4.42, 2.40, 3.19, 3.22, 1.69, 3.28, 3.09, 1.87, 3.15, 4.90, 3.75, 2.43, 2.95, 2.97, 3.39, 2.96, 2.53, 2.67, 2.93, 3.22, 3.39, 2.81, 4.20, 3.33, 2.55, 3.31, 3.31, 2.85, 2.56, 3.56, 3.15, 2.35, 2.55, 2.59, 2.38, 2.81, 2.77, 2.17, 2.83, 1.92.
**D2** The second data set is reported by [28]. It concerns the strengths of 1.5 cm glass fibers, measured at National physical laboratory, England. The data are as follows: 0.55, 0.93, 1.25, 1.36, 1.49, 1.52, 1.58, 1.61, 1.64, 1.68, 1.73, 1.81, 2.00, 0.74, 1.04, 1.27, 1.39, 1.49, 1.53, 1.59, 1.61, 1.66, 1.68, 1.76, 1.82, 2.01, 0.77, 1.11, 1.28, 1.42, 1.50, 1.54, 1.60, 1.62, 1.66, 1.69, 1.76, 1.84, 2.24, 0.81, 1.13, 1.29, 1.48, 1.50, 1.55, 1.61, 1.62, 1.66, 1.70, 1.77, 1.84, 0.84, 1.24, 1.30, 1.48, 1.51, 1.55, 1.61, 1.63, 1.67, 1.70, 1.78, 1.89.

Statistical descriptions of these two data sets are given in Table 6.

**Table 6:** Descriptive statistics for D1 and D2.

	*n*	mean	median	standard deviation	skewness	kurtosis
D1	50	2.95	2.94	0.64	0.40	1.02
D2	63	1.51	1.59	0.32	-0.88	0.80

In particular, we see that the data sets have different skewness features; right skewed for D1 and left skewed for D2.

Then, we compare the fits of the EPGWG model with other competitors also based on the Weibull distribution, namely the odd gamma Weibull-geometric (OGWG) model by [29], beta-Weibull (BW) model by [4], gamma-Weibull (GW) model [29] and the standard Weibull distribution.

The MLEs are computed via the R software, by using the algorithm L-BFGS-B. The minus log-likelihood function is evaluated. Also, well-established goodness-of-fit measures are considered: Akaike Information Criterion (AIC), Cramer-von Mises (W*) and Anderson-Darling (A*). The golden rule is: the lower the values of these criteria, the better the fit. The value for the Kolmogorov-Smirnov (KS) statistic and its p-value are also provided. The efficiency of the maximum likelihood method along with the above criteria is well-established in a data analysis setting. In this regard, we may refer to the works of [30], [31] and [32], and the references therein.

**Fig 6:**
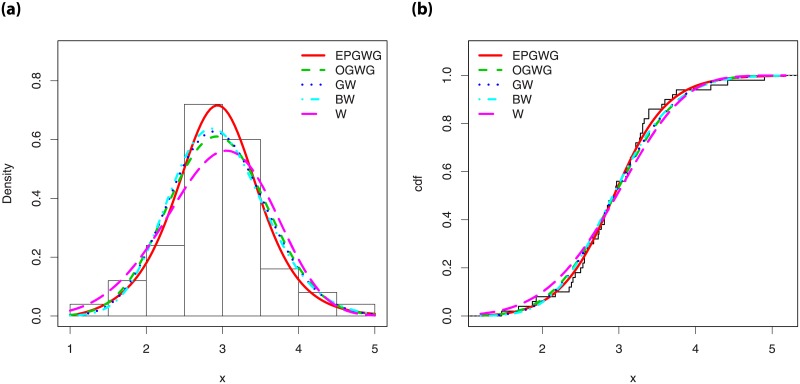
Plots of the (i) estimated pdfs over the histogram and (ii) estimated cdf over the empirical cdf for D1.

**Fig 7:**
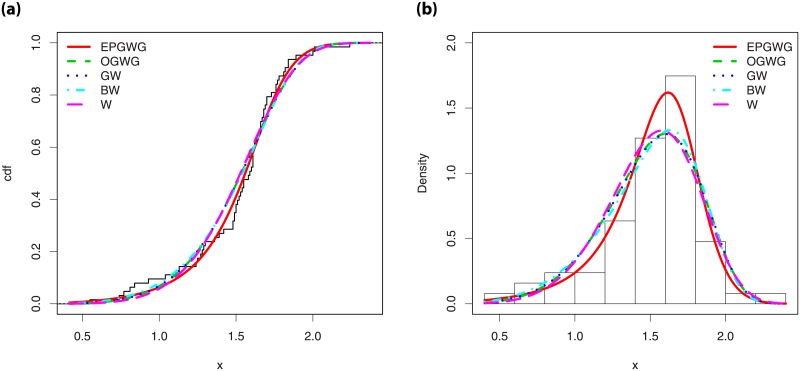
Plots of the (i) estimated pdfs over the histogram and (ii) estimated cdf over the empirical cdf for D2.

Tables 7 and 8 present the MLEs, as well as their standard errors, for D1 and D2, respectively. The values of the considered criteria for the considered models are given in Tables 9 and 10 for D1 and D2, respectively. From these values, it is clear that the EPGWG model is the best: it possesses the lowest AIC, W*, A* and the greatest p-value (K-S) as well. Figs 6 and 7 show the fits of the estimated pdfs over the histograms and estimated cdfs over the empirical cdfs for D1 and D2, respectively. Nice fits for the EPGWG model can be observed.

**Table 7:** MLEs and their standard errors (bottom) for D1.

Model	*α*	*β*	λ	*μ*	*θ*
EPGWG	0.5413 (0.6974)	21.7307 (0.5872)	4.9224 (1.8777)	2.0174 (2.7770)	0.9748 (0.0537)
OGWG	0.7288 (1.1739)	10.3885 (8.6513)	0.6478 (2.4026)	0.6004 (3.1955)	
BW	4.1328 (4.4343)	1.1059 (2.2364)	2.5999 (1.6052)	0.4248 (0.1426)	
GW	0.4043 (0.1880)	15.1849 (9.1757)	4.2117 (7.0929)		
W	4.7824 (0.4801)	0.3121 (0.0097)			

**Table 8:** MLEs and their standard errors (bottom) for D2.

Distribution	*α*	*β*	λ	*μ*	*θ*
EPGWG	1.0816 (1.8981)	0.7213 (0.2897)	0.5339 (0.5678)	3.3051 (1.7041)	0.9464 (0.0772)
OGWG	2.2505 (1.6675)	2.1982 (1.4436)	0.1236 (1.0890)	0.6436 (0.3192)	
BW	0.6335 (0.1348)	0.1997 (0.0323)	6.8846 (0.3042)	0.7530 (0.0027)	
GW	2.1990 (0.8245)	2.1596 (1.0020)	0.6639 (0.1099)		
W	5.7807 (0.5760)	0.6142 (0.0139)			

**Table 9:** Values of the considered criteria for D1.

Model	$-\hat{\ell }$	AIC	W*	A*	KS	p-value (KS)
EPGWG	46.4481	102.8963	0.0358	0.2668	0.0728	0.9536
OGWG	48.0628	104.1257	0.0770	0.5364	0.1071	0.6147
BW	47.6358	103.2717	0.0702	0.4859	0.0955	0.7517
GW	48.8049	103.6098	0.0726	0.5052	0.1002	0.6960
W	50.0858	104.1717	0.1194	0.8114	0.1298	0.3681

**Table 10:** Values of the considered criteria for D2.

Distribution	$-\hat{\ell }$	AIC	W*	A*	KS	p-value (KS)
EPGWG	11.9175	33.8351	0.0885	0.5083	0.0976	0.5847
OGWG	14.8815	37.7662	0.2096	1.1634	0.1469	0.1316
BW	13.8646	35.7292	0.1693	0.9508	0.1404	0.1664
GW	14.8080	35.6160	0.2051	1.1397	0.1460	0.1359
W	14.8815	37.7662	0.2096	1.1634	0.1469	0.1316

## 7 Concluding remarks and perspectives

Based on the exponentiated power generalized Weibull (EPGW) and power series distributions, we introduce a new family of lifetime distributions called the exponentiated power generalized Weibull power series (EPGWPS) family. It has the features to extend several widely used distributions in the literature and to introduce a myriad of new ones, such as the exponentiated power generalized Weibull geometric (EPGWG) distribution. The desirable properties of the family are revealed in this study. In particular, the EPGWG shows a wide panel of monotonic and non-monotonic shapes for the corresponding pdf and hrf, which outperforms the former EPGW distribution in terms of flexibility. We provide some expressions for the quantile function, raw moments, incomplete moments, skewness and kurtosis. By using two practical data sets and standard goodness-of-fit statistics, we show that the EPGWG model is more adequate in fitting these data than some other models based on the Weibull distribution. As perspective of the EPGWPS family, we believe that the also introduced exponentiated power generalized Weibull Poisson (EPGWP_*o*_), exponentiated power generalized Weibull binomial (EPGWB) and exponentiated power generalized Weibull logarithmic (EPGWL) distributions can have suitable issue in various statistical analyzes (data fitting, regression, life testing…). Also, from the perspective of practical applications, the complexity of the EPGWG model could also be studied via Bayesian methods. In particular, one can consider the deviance information criterion available in Bayesian inference, which is deemed as a generalization of the AIC, providing a combined measure of model fit and complexity. In this regard, we refer to the works of [33], [34], [35] and [36]. This aspect needs further investigation that we leave for a future work.

## Supporting information

S1 Fig (EPS)

S2 Fig (EPS)

S1 File (PDF)
