# Supplementary material for: Exponentiated power generalized Weibull power series family of distributions: Properties, estimation and applications
Source: PLoS One. 2020 Mar 20;15(3):e0230004. doi: 10.1371/journal.pone.0230004 (PMC7083325; doi:10.1371/journal.pone.0230004)
Supplement: S1 File — (PDF) [file pone.0230004.s003.pdf]

Supporting information files

February 17, 2020

The following data are used in the paper.

D1 The first data set is obtained from [1]. The data are as follows: 3.70, 2.74, 2.73, 2.50, 3.60, 3.11, 3.27, 2.87, 1.47, 3.11, 4.42, 2.40, 3.19, 3.22, 1.69, 3.28, 3.09, 1.87, 3.15, 4.90, 3.75, 2.43, 2.95, 2.97, 3.39, 2.96, 2.53, 2.67, 2.93, 3.22, 3.39, 2.81, 4.20, 3.33, 2.55, 3.31, 3.31, 2.85, 2.56, 3.56, 3.15, 2.35, 2.55, 2.59, 2.38, 2.81, 2.77, 2.17, 2.83, 1.92.

D2 The second data set is reported by [2]. It concerns the strengths of 1.5 cm glass fibers, measured at National physical laboratory, England. The data are as follows: 0.55, 0.93, 1.25, 1.36, 1.49, 1.52, 1.58, 1.61, 1.64, 1.68, 1.73, 1.81, 2.00, 0.74, 1.04, 1.27, 1.39, 1.49, 1.53, 1.59, 1.61, 1.66, 1.68, 1.76, 1.82, 2.01, 0.77, 1.11, 1.28, 1.42, 1.50, 1.54, 1.60, 1.62, 1.66, 1.69, 1.76, 1.84, 2.24, 0.81, 1.13, 1.29, 1.48, 1.50, 1.55, 1.61, 1.62, 1.66, 1.70, 1.77, 1.84, 0.84, 1.24, 1.30, 1.48, 1.51, 1.55, 1.61, 1.63, 1.67, 1.70, 1.78, 1.89.

References

[1] Nichols, M.D. and Padgett, W.J. (2006). A bootstrap control chart for Weibull percentiles, Qual. Reliab. Eng. Int., 22, 141-151.

[2] Smith, R.L. and Naylor, J. (1987). A comparison of maximum likelihood and Bayesian estimators for the three-parameter Weibull distribution, Journal of the Royal Statistical Society: Series C (Applied Statistics), 36, 3, 358-369.
